# Validation of Teleconference-based Goniometry for Measuring Elbow Joint Range of Motion

**DOI:** 10.7759/cureus.6925

**Published:** 2020-02-09

**Authors:** Paul A Dent, Benjamin Wilke, Sarvram Terkonda, Ian Luther, Glenn G Shi

**Affiliations:** 1 Physics, Hampden-Sydney College, Farmville, USA; 2 Orthopaedics, Mayo Clinic, Jacksonville, USA; 3 Plastic Surgery, Mayo Clinic, Jacksonville, USA; 4 Physical Therapy, Mayo Clinic, Jacksonville, USA

**Keywords:** elbow, range of motion, rom, orthopedics, physical therapy, goniometry, telemedicine

## Abstract

Background

Range of motion (ROM) is a critical component of a physician’s evaluation for many consultations. The purpose of this study was to evaluate if teleconference goniometry could be as accurate as clinical goniometry.

Methods

Forty-eight volunteers participated in the study. There was a sample size of 52 elbows. Each measurement was recorded consecutively in person, through teleconference, and still-shot photography by two researchers trained in goniometry. Measurements of maximum elbow flexion and extension were taken and recorded.

Results

Teleconference goniometry had a high agreement with clinical goniometry (Pearson coefficient: flexion: 0.93, Extension: 0.87). Limits of agreement found from the Bland-Altman test were 7⁰ and -3⁰ for flexion and 10.4⁰ and -7.4⁰ for extension. A t-test revealed a P-value of less than 0.001 between teleconference and clinical measurements, proving the data are significant.

Conclusions

ROM measurements through a teleconferencing medium are comparable to clinical ROM measurements. This would allow for interactive elbow ROM assessment with the orthopedist without having to incorporate travel time and expenses.

## Introduction

The increasing cost of healthcare can lead to a gap in a patient’s ability to access proper care. To account for this crisis, hospitals in Europe and Australia have experimented with telemedicine [1-4]. Telemedicine is a cost-effective way to consult with patients from their own home, eliminating travel expenses and time while providing care [2]. Using Telemedicine to determine ROM has peaked the interests of many physicians [1-7]. ROM goniometry is a vital component of an orthopedic surgeon’s examination. Measurements can be used to establish a baseline for patients, guide further improvement, or for a post-operation comparison [5,7-8].

 Previous studies have shown promising results in validating telemedicine using smartphone photography to provide measurements within the acceptable error range of a goniometer for fingers and elbows [5,7]. There is also a strong agreement between telehealth and in-person clinical visits with respect to diagnoses of patients with chronic musculoskeletal conditions [4]. This is also important as photography-based goniometry relied less on observer expertise than clinical goniometry [6].

 Although previous research has shown strong findings in digital photography, none have validated ROM through a teleconference [5-7]. Teleconference will allow for a real-time measurement where photography may lead to excess waiting.

 The purpose of this study is to determine if teleconferencing can be used as an alternative to evaluate ROM. This study could provide an increase in physician accessibility for patients in remote areas. 

## Materials and methods

All volunteers were over the age of 18 years and in healthy condition. The volunteer must be able to comfortably perform flexion and extension of the elbow without pain. The volunteer was excluded if they had a previous or ongoing injury. If the volunteer was uncomfortable with teleconferencing they were excluded from participation.

Forty-eight healthy volunteers were recruited to participate in this prospective study. The study took place in a clinical setting to have standardized conditions. Every volunteer was asked to perform full flexion and extension of the elbow joint. The joint range of motion was measured and recorded in-person by a research personnel trained in goniometry by a board-certified physical therapist. The research personnel, blinded to their prior results, asked the patient to repeat the full extension and flexion of the same joints but through a teleconferencing medium. The research personnel recorded the goniometric measurements through the teleconferencing system and recorded the data. Finally, screen photography of the joints was measured by a second research personnel to determine interobserver reliability. The second researcher was blinded to the results of the first measurements.

Clinical goniometry

**Figure 1:**
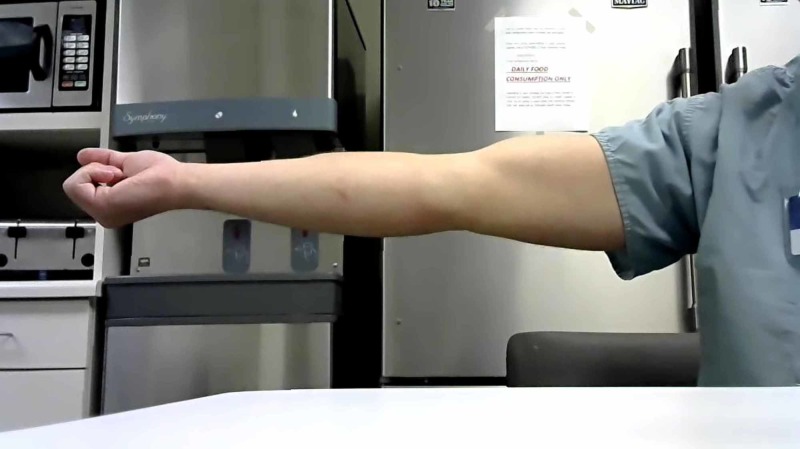
Maximum extension A participant demonstrates maximum extension during a clinical trial

**Figure 2:**
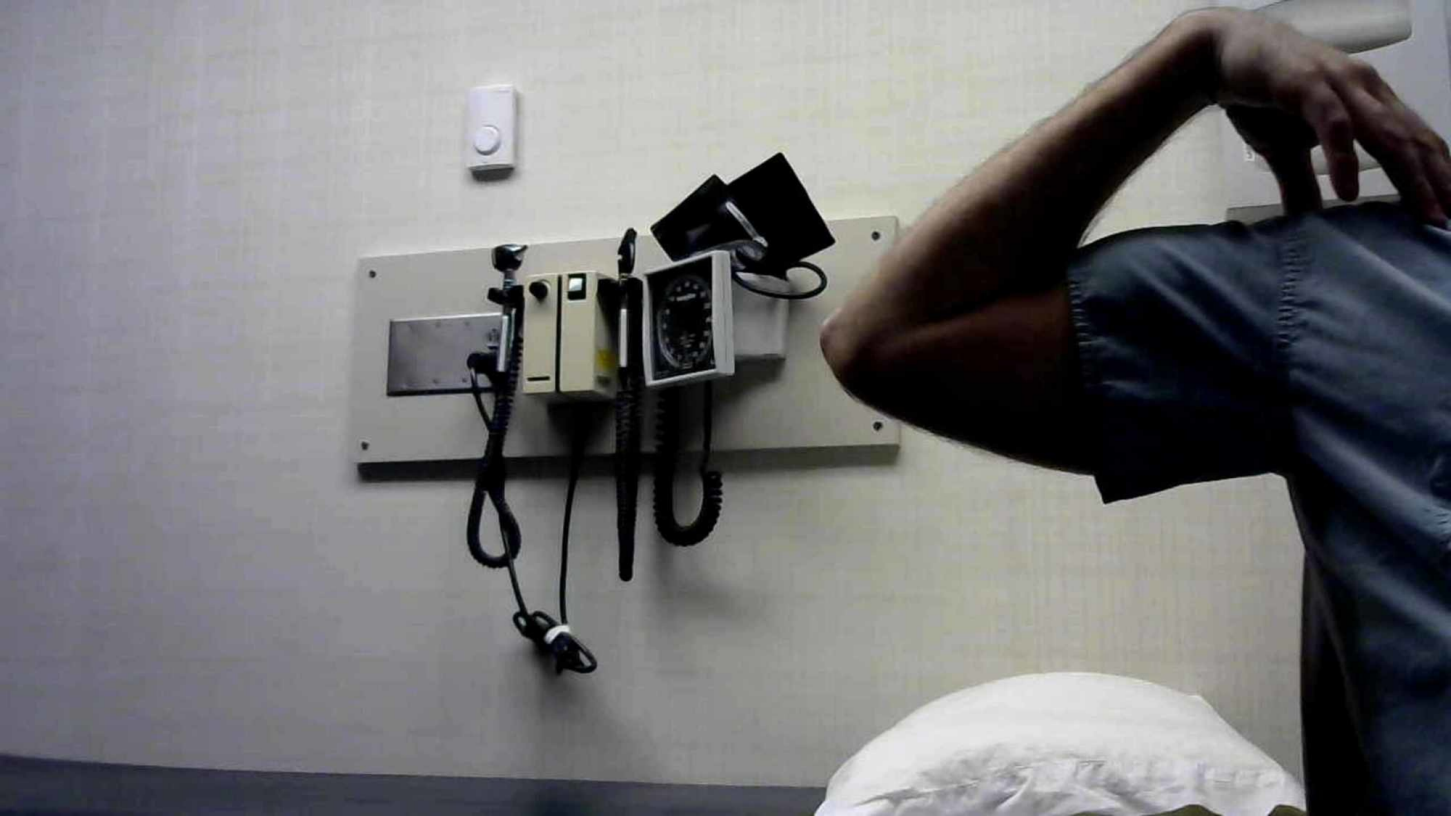
Maximum flexion A participant demonstrates maximum flexion during a clinical trial

The researcher measured maximum flexion and extension of the elbow using a standard goniometer. The position of the elbow during the experiment was standardized for all participants: the participants were recorded standing with elbows extended with the palms of the hand fully supinated (Figure 1). For flexion measurements, the participant was instructed to attempt to place their hand on their shoulder (Figure 2). The researcher used their preferred landmarks to record the measurements.

Telemedical goniometry

Full flexion and extension of the elbow using the same goniometer that was used for clinical goniometry. Participants were measured through a computer-mounted camera via teleconference. Participants were positioned 3-5 feet from the web camera. The camera used was a Logitech C270 720-pixel camera (Logitech, Newark, CA, USA). Each were informed to achieve maximum extension perpendicular to the web camera with palms fully supinated and recorded the ROM by placing the goniometer up to the computer screen (Figure 1). Once recorded, the researcher took a screenshot of the teleconference to emulate digital photography for the second researcher to record. This process was repeated for maximum elbow flexion using the same method in the clinical trial (Figure 2).

Still-shot photography

The second research personnel blinded to the data recorded by the first used the same goniometer from the previous trials. The researcher measured the full flexion and extension of the participants ROM photographs and recorded the data.

Statistical analysis

A paired two-sample for means t-test was performed to determine the significance of the data. The test calculated a sample size of 52 measurements based on a mean difference of 5⁰, an α of 0.05 (Table 1 & Table 2).

**Table 1:** t-Test: clinical vs. telemedical goniometry This table represents in-depth statistics for the comparison between clinical goniometry and telemedicine-based goniometry

Flexion	Clinic	Teleconference	Extension	Clinic	Teleconference
Mean	41.50	39.46	Mean	0.92	1.48
Variance	44.37	40.88	Variance	12.50	19.08
Observations	52.00	52.00	Observations	52.00	52.00
Pearson Correlation	0.93		Pearson Correlation	0.87	
Hypothesized Mean Difference	5.00		Hypothesized Mean Difference	5.00	
df	51.00		df	51.00	
t Stat	-8.50		t Stat	-18.29	
P(T<=t) one-tail	1.2E-11		P(T<=t) one-tail	2.4E-24	
t Critical one-tail	1.68		t Critical one-tail	1.68	
P(T<=t) two-tail	2.4E-11		P(T<=t) two-tail	4.7E-24	
t Critical two-tail	2.01		t Critical two-tail	2.01	

**Table 2:** t-Test: clinical vs. photography goniometry This table represents in-depth statistics for comparing clinical vs. photography-based goniometric measurements

Flexion	Clinic	Photography	Extension	Clinic	Photography
Mean	41.50	40.02	Mean	0.92	0.38
Variance	44.37	25.90	Variance	12.50	4.75
Observations	52.00	52.00	Observations	52.00	52.00
Pearson Correlation	0.73		Pearson Correlation	0.82	
Hypothesized Mean Difference	5.00		Hypothesized Mean Difference	5.00	
df	51.00		df	51.00	
t Stat	-5.57		t Stat	-14.93	
P(T<=t) one-tail	4.7E-07		P(T<=t) one-tail	1.5E-20	
t Critical one-tail	1.68		t Critical one-tail	1.68	
P(T<=t) two-tail	9.4E-07		P(T<=t) two-tail	3.0E-20	
t Critical two-tail	2.01		t Critical two-tail	2.01	

**Figure 3:**
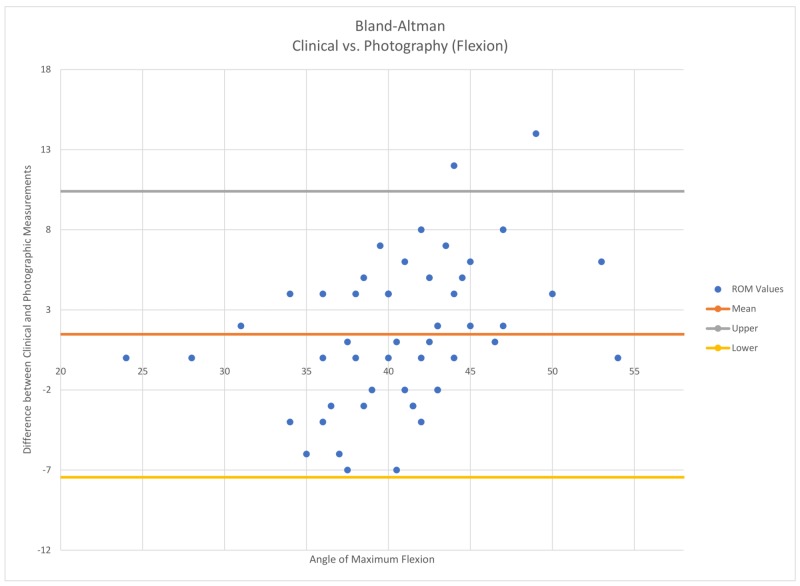
Clinical vs. photography A Bland–Altman plot representing flexion comparison measurements that fell within the 95% confidence interval

**Figure 4:**
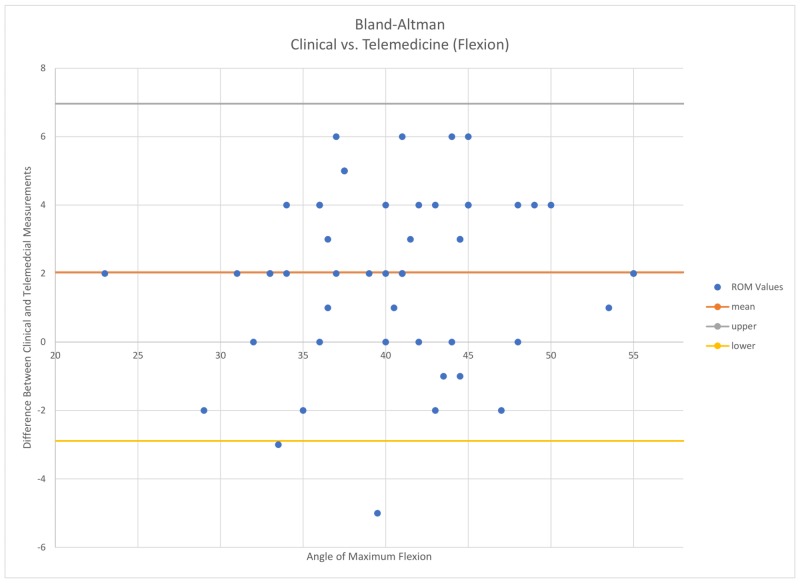
Clinical vs. telemedicine A Bland–Altman plot representing the amount of measurements that fell within a 95% confidence interval

Interobserver reliability between clinical, photo, and teleconferencing was calculated using Pearson coefficients for all measurements. An intraclass correlation coefficient (ICC) less than 0.4 represents low agreement, an ICC between 0.4 and 0.59 represents fair agreement, an ICC between 0.6 and 0.75 represents a good agreement, and an ICC above 0.75 represents exceptional agreement between measurements [7]. A Bland-Altman analysis was also performed to determine the limits of agreement between clinical and teleconferencing measurements (Figure 3 and Figure 4).

## Results

Forty-eight subjects and 52 measurements were recorded in this study. The average clinical goniometry measurements resulted in flexion 41.5 +/- 6.7 degrees and extension 0.93 +/- 3.5 degrees. Teleconference measurements held similar results with flexion 39.5 +/- 6.4 degrees and extension 1.5 +/- 4.4 degrees while photography-based measurements were 40 +/- 5.1 and 0.4 +/- 2.2 degrees. The differences recorded between measurements were statistically significant between clinical and photo as well as between clinical and teleconferencing. There was a mean difference of 2.7 +/- 1.7 (paired t-test, P < .0001) degrees in flexion between clinical and teleconferencing measurements. The mean difference between clinical and photography-based measurements were 3.7 +/- 3 (paired t-test, P < .0001) degrees for flexion. The findings are similar for extension (Table 1 & Table 2 for in-depth statistics).

Interobserver reliability

All measurements represented strong reliability. Clinical vs videoconferencing yielded a Pearson coefficient of 0.93 for flexion and 0.86 for extension. Clinical vs. photography yielded a Pearson coefficient of 0.73 for flexion and 0.82 for extension. The Bland-Altman test (Figure 3 and Figure 4) revealed that 50 out of 52 of the total flexion measurements fell within the limits of agreement (95% confidence interval) for telemedicine and clinical goniometry. Clinical vs. photographic yielded the same results. 

## Discussion

This study validated that goniometric ROM measurements over a teleconferencing medium are consistent with clinical measurements. Teleconferencing measurements, like photography also required less skill than taking a ROM measurement in person [6].

 Patients could have a teleconference with a physician without needing to travel to the clinic to evaluate ROM. This may translate to cost savings for our medical systems [2]. This study may also improve patient return rate as they may be more likely to follow up with a physician since there is no need for travel.

 Previous studies have reported accuracy in photography-based ROM measurements yet none have attempted to validate ROM measurements through a teleconferencing medium [5-7]. This is important because a video consultation with a physician would allow the patient to have their questions answered in real time. Photography has been proven accurate; however, it may lead to excess waiting for the patients and ultimately decrease satisfaction.

 Teleconferencing has been reported to be satisfactory for patients with chronic musculoskeletal conditions and virtual outreach consultations [3-4]. Dermatology has been a front-runner in the use of telemedicine along with optometry. This study can increase the uses for telehealth in the orthopedic field. Patients are more likely to return for follow-visits and physical therapy appointments if the location is closer to home. Thus, it is expected that this percentage may be higher for telehealth as it requires no travel at all. It would make life easier for seniors or those recovering from arthroplasties. This study would also benefit rural communities by providing easy access to physicians who may have been out of reach prior to the adoption of teleconference.

 The limitations of this study include the lack of measurers and the ability of being tech-savvy. With telemedicine, patients must be able to understand how to use the system to speak with the physician and must be connected to the internet. There was only one measurer for clinical ROM measurements and teleconference measurements. There was also one researcher measuring all the photography-based ROM measurements. Although every measurement taken was standardized and unbiased, it may be beneficial to include other researchers trained in goniometry to further strengthen the findings.

 Video conferencing measurements tended to underestimate the ROM values compared to the clinical setting. This could be explained by the difficulty to identify the “bony” landmarks without feeling the patients' elbow. The photography-based measurements had an average difference of 3.7 degrees compared to the videoconference with an average difference of 2.7 degrees. This could be because the researchers used slightly different landmarks when recording their ROM measurements. Although there was a greater difference, it was still under the accepted value of 5 degrees [5, 8].

## Conclusions

Teleconference can be a reliable resource for evaluating elbow ROM (difference between maximum flexion and extension). Our findings demonstrated acceptable angular measurements (maximum elbow flexion and extension) via teleconference screen. Results were similar to still photograph and clinical goniometer. The findings of this study may help lead to validating ROM measurements of other joints through a teleconferencing medium. 
